# Fertility decision of Iranian women during the COVID-19 pandemic and home quarantine: A cross-sectional study in Iran

**DOI:** 10.3389/fpsyg.2022.993122

**Published:** 2022-11-15

**Authors:** Poorandokht Afshari, Parvin Abedi, Maryam Beheshtinasab

**Affiliations:** Midwifery Department, Reproductive Health Promotion Research Center, Ahvaz Jundishapur University of Medical Sciences, Ahvaz, Iran

**Keywords:** COVID-19, pandemic, fertility decision, home quarantine, Iran

## Abstract

**Background:**

Fertility decision is defined as the mutual decision of partners toward having children, which may be equally controlled by the two partners or dominantly powered by the female partner. This study aimed to evaluate fertility decision of women during the COVID-19 pandemic in Iran.

**Methods:**

This cross-sectional study was conducted on 600 women (300 pregnant and 300 non-pregnant) during the COVID-19 pandemic. A demographic questionnaire and the Attitudes toward Fertility and Childbearing Scale were used to collect the data. Independent t-test, Chi-square test, and logistic regression were used for analyzing data.

**Results:**

The mean ± SD age of participants with negative and positive attitude toward fertility was 28.96 ± 5.88 and 28.39 ± 6.2, respectively. Almost half of the studied women postponed their pregnancy to after the pandemic. The total score of fertility decision in women with positive attitudes toward fertility was 95.43 ± 18.51 compared to 46.73 ± 17.2 in women with negative attitudes toward fertility (*p* < 0.0001). None-employed women had 0.595 times the odds of having a positive attitude toward fertility (95% CI: 0.423–0.836). Women who were not pregnant had 1.5 times the odds of having a positive attitude toward childbearing (95% CI: 1.067–2.112). In addition, women who were not hospitalized during the pandemic had 0.520 times the odds of having a positive attitude toward fertility (95% CI: 0.342–0.790).

**Conclusion:**

The results of this study showed that half of the women postponed their pregnancy to after the pandemic. Also, employed women, women who were not pregnant, and women who were not hospitalized during pandemic were more likely to have positive attitudes toward fertility during the COVID-19 pandemic. Policymakers should devise some strategies to clarify the benefits and harms of pregnancy during crises such as COVID-19 pandemic.

## Introduction

Fertility decision is defined as mutual decision of partners toward having children, which may be equally controlled by the two partners or dominantly powered by the female partner (Stein et al., 2014). Factors such as age, education, economic status, and desired number of children can influence fertility decision of couples (Adhikari, 2010). While factors such as higher educational attainment, availability of contraceptive methods, and Gross Domestic Products (GDP) may be negatively correlated with fertility decision, religion seems to have a positive impact on it (Götmark and Andersson, 2020). Iran is a country located in the Middle East and has a population of 83,992,949 (Worldometer, 2022a). Evidence shows that fertility rate is under replacement in Iran (1.5 children per woman during reproductive age; Hosseini et al., 2021). A recent study in Iran showed that those participants with a higher age-gap with their husbands and with having more children had more negative attitudes toward fertility, and those who had husbands with higher level of education, and higher score of social and emotional factors were more positive toward fertility (Jafarzadeh Esfehani et al., 2019).

Like all other countries across the globe, Iran faced the COVID-19 pandemic in early 2020. According to statistics, 7.2 million people have so far been affected with COVID-19 infection and 141,000 individuals have died from this infection (Worldometer, 2022b). In addition to mortality, the COVID-19 pandemic has changed many aspects of everyday life including couples’ fertility decisions. For example, according to Albeitawi et al., around 80% of Jordanian women believed that pregnancy during the pandemic is dangerous, and infertile women did not like to use assisted reproductive technologies for pregnancy (Albeitawi et al., 2022). Also, Ghaznavi et al. found that the number of births in Japan declined during the pandemic (Ghaznavi et al., 2022). No study has directly addressed fertility decision of couples during the COVID-19 pandemic in Iran, a study by Peivandi et al. reported that Iranian infertile couples were not willing to continue their infertility treatment because of fear of transmission of infection to the fetus (Peivandi et al., 2022). Confirming the results of Peivandi et al., Kamath et al. in India found that almost one-third of infertile couples paused their treatment and postponed it until after pandemic (2021). Also, another study in the United States showed that out of 16,000 couples who decided to conceive before COVID-19 pandemic, almost 70% decided to postpone their pregnancy temporarily (Malloy and Bradley, 2021). Given the insufficient information regarding fertility decision during COVID-19 pandemic in Iran, this study was conducted to evaluate fertility decision and its effective factors among Iranian reproductive-aged women during the COVID-19 pandemic.

## Materials and methods

This was a cross-sectional study conducted on 600 (300 pregnant and 300 non-pregnant women) during the COVID-19 pandemic. The design of this study was approved by the Ethics Committee of Ahvaz Jundishapur University of Medical Sciences (Ref. ID: IR.AJUMS.REC.1400.334). All women provided written informed consent prior to data collection, and participants were assured about confidentiality of data.

### Inclusion/exclusion criteria

Eligible participants were pregnant or non-pregnant monogamous women in reproductive age (18–40 years), who had basic literacy. Women with a history of infertility and medical contraindication for pregnancy were excluded from the study.

### Setting

The public health centers are classified into the west and east banks of Karun River in Ahvaz city, and in this study, three centers from each bank were chosen. Eligible women in these centers received a phone call and then a link to access a website that was provided for them.

### Sampling

The following formula was used for sampling:


$$n=\frac{Z_{1-\alpha /2}^{2}\times p\left(1-p\right)}{d^{2}}$$

1−α/2 = 1.96, *p* = 50%, *d* = 0.4, *N* = 600.

### Data collection tools

A demographic questionnaire and the Attitudes toward Fertility and Childbearing Scale were used to collect the data. The demographic questionnaire included questions regarding age, occupation, education, age, husband’s occupation and educational attainment, number of children, infection with COVID-19, hospitalization, and vaccination.

The Attitudes toward Fertility and Childbearing Scale (AFCS) was initially developed by Soderberg et al. and has 27 questions in three subscales including the importance of fertility for the future, childbearing as a barrier at the present time, and social identity (Soderberg et al., 2013). It is scored based on a 5-item Likert scale, with 5 indicating “strongly agree” and 1 indicating “strongly disagree.” A higher score indicates more positive attitudes toward fertility and childbearing.

The psychometric properties of the Persian version of this questionnaire were assessed by Baezzat et al. (2017). During the psychometric evaluation of this questionnaire in Iran, the number of questions was reduced to 23, and the number of subscales rose to four, namely “children as a pillar of life (questions 1, 2, 3, 4, 6, 8, 21, and 23),” child as an obstacle (questions 13, 14, 15, 16, 17, 18),” “postponing fertility to future (questions 5, 7, 9, 11, 12),” and “fertility after fulfilment of preconditions (for example completing education or saving money, questions 10, 19, 20, and 22).” It is worth mentioning that questions 9–20 are scored negatively. The maximum and minimum scores of this questionnaire are 115 and 23, respectively. Because questionnaire does not have a cutoff point, we classified women according to the mean of the scores. We considered scores more than the mean of AFCS as indicating “positive attitudes toward fertility” and those below the mean indicating “negative attitudes toward fertility.”

### Procedure

The phone numbers of eligible women were obtained from the six public health centers. The women subsequently received a phone call, and in case they were willing, they were requested to complete two questionnaires on a web page. The first page before the questionnaires start was dedicated to informed consent. Women were requested to sign this form before answering the questions. One of the researchers (MB) was available in case the participants had any question. Data collection started in September 2021 and was completed in March 2022.

### Statistics

All data were entered into SPSS version 23. Normal distribution of continuous data was evaluated using Shapiro–Wilk test. Independent t-test and Chi-square tests were used for comparing continuous and categorical data, respectively. Logistic regression was used to test the association between attitudes toward fertility and other variables. *p* < 0.05 was considered statistically significant.

### Results

We invited 750 women to participate in this study, of which 150 of them did not respond, or did not complete questionnaires, and 600 (300 pregnant and 300 non-pregnant women) returned completed questionnaires. Table 1 shows the demographic characteristics of the participants with negative and positive attitudes toward childbearing during the COVID-19 pandemic. As evident from this table, the two groups did not show any significant difference regarding age, age of husband, length of marriage, and number of children. A significantly higher rate of employment and significantly lower rates of pregnancy and hospitalization due to COVID-19 were observed in women with positive attitudes toward childbearing. Apart from these, the two groups did not have any other significant differences regarding other variables.

**Table 1:** Demographic characteristics of two groups with positive and negative attitudes toward pregnancy and childbearing.

Variables	Negative attitude toward pregnancy *n* = 331	Positive attitude toward pregnancy *n* = 269	*p* Value
	Mean ± SD or *N* (%)		
Age (year)	28.96 ± 5.88	28.39 ± 6.2	0.244
Age of husband (year)	29.96 ± 5.57	29.55 ± 5.82	0.914
Living child	0.91 ± 0.92	0.89 ± 0.99	0.22
Length of marriage (year)	4.51 ± 3.22	4.40 ± 3.2	0.660
Number of children	0.92 ± 0.99	0.87 ± 0.91	0.516
**Education**			
High school	80 (24.2)	63 (23.4)	0.977
Diploma	195 (58.9)	160 (59.5)	
University degree	56 (16.9)	46 (17.1)	
**Occupation**			
Housewife	172 (52)	102 (37.9)	<0.0001
Employee	159 (48)	167 (62.1)	
**Education of husband**			
High school	69 (20.8)	57 (21.2)	0.290
Diploma	190 (57.4)	167 (62.1)	
University degree	72 (21.8)	45 (16.7)	
**Occupation of husband**			
Un-employed	38 (11.5)	31 (11.5)	0.055
Self-employed	115 (34.7)	87 (32.3)	
Employed	178 (53.8)	151 (56.2)	
**Economic status**			
Good	60 (18.1)	58 (21.6)	0.475
Moderate	179 (54.1)	145 (53.9)	
Poor	92 (27.8)	66 (24.5)	
**Affected with COVID-19 disease**			
Yes	182 (55)	135 (50.2)	0.138
No	149 (45)	134 (49.8)	
**Admission to hospital**			
Yes	113 (34.1)	63 (23.4)	0.003
No	218 (65.9)	206 (76.6)	
**Do you find COVID-19 dangerous?**			
Yes	176 (53.2)	156 (58)	0.136
No	155 (46.8)	113 (42)	
**Have you had vaccination?**			
Yes (1 dose)	71 (21.5)	54 (20.1)	0.507
Yes (2 doses)	92 (27.8)	85 (31.6)	
No	168 (50.7)	130 (48.3)	
**Pregnancy during pandemic**			
Yes	183 (55.2)	117 (43.5)	0.003
No	148 (44.7)	152 (56.5)	

Table 2 shows the views of participants in the two groups of negative and positive attitudes toward fertility according to AFCS questionnaire. As this table shows, women with a positive attitude toward pregnancy and childbearing were more likely to consider a “child as a pillar of life,” and were less likely to consider a “child as an obstacle” or to be considering “postponing fertility to future” or “after fulfillment of preconditions” (*p* < 0.0001). The total score of fertility decision in women with positive attitude toward fertility was 95.43 ± 18.51 compared to 46.73 ± 17.2 in women with negative attitude toward fertility (*p* < 0.0001).

**Table 2:** Comparison of scores of fertility decision among two groups with positive and negative attitudes toward pregnancy and childbearing during the COVID-19 pandemic.

Components	Negative toward pregnancy *n* = 331	Positive toward pregnancy *n* = 269	*p* Value
	Mean ± SD		
The child as a pillar of life	11.36 ± 6.53	46.98 ± 9.34	<0.0001
Child as an obstacle	12.69 ± 5.59	23.68 ± 4.66	<0.0001
Postponing fertility to the future	6.68 ± 2.80	16.21 ± 2.40	<0.0001
Fertility after fulfilment of preconditions	15.81 ± 2.28	8.56 ± 2.11	<0.0001
Total scores	46.73 ± 17.2	95.43 ± 18.51	<0.0001

Table 3 shows the reasons for lack of interest in childbearing during the COVID-19 pandemic among the two groups with negative and positive attitudes toward pregnancy. The two groups did not show any significant difference regarding factors such as inappropriate social conditions during the pandemic (losing job, or income reduction), the unknown effect of the COVID-19 infection on the fetus and the pregnant mother, higher probability of complications and death of the pregnant mother in case of getting infected, reluctance to be vaccinated during pregnancy, no adequate quarantine during pregnancy due to employment, and the risk of pregnancy during the COVID-19 pandemic.

**Table 3:** Reasons for lack of interest in pregnancy during the COVID-19 pandemic among two groups with negative and positive attitudes toward pregnancy.

Reasons	Negative toward pregnancy *n* = 331	Positive toward pregnancy *n* = 269	*p* Value
	*N* (%)		
Inappropriate social conditions during the pandemic	75 (22.7)	56 (20.8)	0.48
The unknown effect of the COVID-19 infection on the fetus and pregnant mother	33 (10)	21 (7.8)	0.39
More possibility of complications and death of the pregnant mother in case of infection	20 (6)	18 (6.7)	0.43
Reluctance to be vaccinated during pregnancy	40 (12.1)	29 (10.8)	0.70
Being employed does not allow adequate quarantine during pregnancy	149 (45)	127 (47.2)	0.62
Pregnancy is not dangerous during the COVID-19 pandemic	14 (4.2)	18 (6.7)	0.20

For a better understanding of the relationship between attitudes toward childbearing, logistic regression was used, and the results are presented in Table 4. As this table shows, none-employed women had 0.595 times the odds of having a positive attitude toward fertility (95% CI: 0.423–0.836). Women who were not pregnant had 1.5 times the odds of having a positive attitude toward childbearing (95% CI: 1.067–2.112). In addition, women who were hospitalized during the pandemic had 0.520 times the odds of having a positive attitude toward fertility (95% CI: 0.342–0.790).

**Table 4:** Logistic regression for assessing the relationship between demographic characteristics and having a positive attitude toward pregnancy and childbearing.

Model	Odds ratio	95% CI	
		Lower	Upper
None employed vs. employed	0.595	0.423	0.836
No Pregnancy during pandemic vs. pregnancy during pandemic	1.501	1.067	2.112
Good Economic status vs. Poor	1.294	0.788	2.126
Moderate Economic status vs. Poor	1.030	0.690	1.538
One dose Vaccination vs. no vaccination	0.958	0.617	1.486
Two doses Vaccination vs. no vaccination	1.006	0.678	1.493
No hospitalization vs. hospitalization	0.520	0.342	0.790
Affected with COVID-19 infection vs. not affected with infection	1.009	0.683	1.472

## Discussion

This study was designed to evaluate the fertility decision of Iranian women in the reproductive age during the COVID-19 pandemic. The results of the present study showed that almost half of the studied women postponed their pregnancy to after the pandemic, which is consistent with Chu et al. in China, who found that fertility intention of 47.7% of women was affected by COVID-19 outbreak (Chu et al., 2022). Results of a study by Albeitawi et al. on 814 fertile and infertile women in Jordan showed that 58.7% of fertile and 76.6% of infertile women believed that pregnancy during pandemic is dangerous (Albeitawi et al., 2022), which is consistent with our results. Furthermore, Kahn et al. who evaluated 1,179 mothers of young children during COVID-19 pandemic found that around 40% of these women postponed their pregnancy to after the pandemic (Kahn et al., 2021) which is in line with our study.

Contrary to our results, however, a study in sub-Saharan, Kenya showed that almost 85% of participants insisted on their fertility decision during the COVID-19 pandemic (Zimmerman et al., 2022). The reason for this discrepancy may be due to the fact that COVID-19 virus in sub-Saharan Africa may not be regarded as dangerous as it is in other countries. For example, a study by Osuagwu et al. (2022) showed that 72.2 and 84.5% of sub-Saharan people were resistant to or hesitant about COVID-19 vaccination, respectively.

According to our results, employed and non-pregnant women had a positive attitude toward fertility. Although employment was not assessed in other studies, a study by Fakehi et al. (2021) showed that nulliparous Iranian women with higher level of education, and better economic status had negative attitude toward fertility during pandemic that is not in line with our results. The reason for this discrepancy may be due to the fact that Fakehi et al. (2021) only recruited nulliparous women, while we recruited nulliparous, multiparous, and pregnant women.

Our results indicated that women who were not hospitalized were more positive toward fertility.

In line with our results, Akinyemi et al. (2022) in a study on 774 married and non-pregnant women found that COVID-19 pandemic caused women to postpone their pregnancy, and this decision was related to age, higher education, and household food insecurity.

Evidence shows that females and those who are suspicious of having COVID-19 disease were more likely to be afraid of the disease (Quadros et al., 2021). Studies show that women generally are more fearful and have more negative expectations about the consequences of a pandemic compared with men (Alsharawy et al., 2021).

Our results showed that pregnant women have more negative attitudes toward childbearing. A qualitative study by Sahin and Kabakci (2021) in Turkey showed that pregnant women during pandemic experienced more fear and anxiety, which create exerts negative emotional effects on pregnant women.

Our results also showed that women with a positive attitude obtained significantly higher scores from the subscales of “considering children as a pillar of life,” having child as an obstacle, “postponing pregnancy to future,” and “fertility after fulfilment of preconditions,” and a higher total score of fertility decision compared to women with a negative attitude toward fertility during the COVID-19 pandemic.

In conclusion, women who had positive attitudes toward fertility during pandemic were willing to postpone their pregnancy to the future, but they believed that a child is a pillar of life.

### Strengths and limitations of the study

This is the first scholarly attempt to evaluate the opinion of women (pregnant and non-pregnant) toward fertility during COVID-19 pandemic in Iran. Despite its merits, this study has some limitations. First, we did not recruit women randomly, and this may affect the generalizability of the study. Second, we did not assess the opinion of husbands about fertility decision. Future studies may wish to consider the view-points of husbands in this regard.

## Conclusion

The results of this study showed that half of the studied women postponed their pregnancy to after the pandemic. Also, employed women, women who were not pregnant, and women who were not hospitalized during the pandemic were more likely to have positive attitudes toward fertility during COVID-19 pandemic. Policymakers should pursue strategies to clarify the benefits and harms of pregnancy during crises such as the COVID-19 pandemic.

## Data availability statement

The raw data supporting the conclusions of this article will be made available by the authors, without undue reservation.

## Ethics statement

The studies involving human participants were reviewed and approved by the Ethics Committee of Ahvaz Jundishapur University of Medical Sciences (Ref. ID: IR.AJUMS.REC.1400.334). The patients/participants provided their written informed consent to participate in this study.

## Author contributions

All authors equally contributed to the conception of the study. MB collected the data. All authors were involved in data analysis and interpretation. PaA prepared the manuscript. All authors contributed to the article and approved the submitted version.

## Conflict of interest

The authors declare that the research was conducted in the absence of any commercial or financial relationships that could be construed as a potential conflict of interest.

## Publisher’s note

All claims expressed in this article are solely those of the authors and do not necessarily represent those of their affiliated organizations, or those of the publisher, the editors and the reviewers. Any product that may be evaluated in this article, or claim that may be made by its manufacturer, is not guaranteed or endorsed by the publisher.
